# Moisture Sorption Isotherms of Fructooligosaccharide and Inulin Powders and Their Gelling Competence in Delaying the Retrogradation of Rice Starch

**DOI:** 10.3390/gels11100817

**Published:** 2025-10-12

**Authors:** Bing Dai, Ruijun Chen, Zheng Wei, Jianzhang Wu, Xingjun Li

**Affiliations:** 1 College of Grain and Strategic Reserves, Henan University of Technology, Zhengzhou 450001, China; bingdai3@126.com (B.D.); qlsszz@126.com (J.W.); 2 Academy of National Food and Strategic Reserves Administration, National Engineering Research Center for Grain Storage and Transportation, Beijing 102209, China; ruijun_chen@126.com (R.C.); wz@ags.ac.cn (Z.W.); 3 College of Food Science and Engineering, Wuhan Polytechnic University, Wuhan 430048, China

**Keywords:** fructooligosaccharide (FOS), inulin, moisture desorption isotherm, hysteresis, seven-parameter polynomial equation, setback viscosity

## Abstract

The accurate determination of the equilibrium moisture content (EMC) of gel-related powdery samples requires strictly controlled conditions and a long time period. In this study, the adsorption and desorption isotherms of two fructooligosaccharide (FOS) powders and three inulin powders were determined using a dynamic moisture sorption analyzer at 0.1–0.9 water activity (a_w_) and 20–35 °C, respectively. The adsorption and desorption isotherms all exhibited type IIa sigmoidal curves; the desorptive isotherm was smooth, the FOS adsorption curves had three inflection points, and the inulin adsorption curves had five inflection points. Large hysteresis between the adsorption and desorption isotherms occurred at 0.1–0.7 a_w_ for FOS and 0.1–0.6 a_w_ for inulin. Seven equations, Boquet, Ferro–Fontan, Guggenheim–Anderson–de Boer (GAB), Generalized D’Arcy and Watt (GDW), modified GAB (MGAB), Peleg, and our developed Polynomial, were found to fit the isotherms of the FOS and inulin samples; for adsorption, the best equations were Ferro–Fontan and GDW, and for desorption, the best equations were Polynomial and MGAB. The GDW and MGAB equations could not distinguish the effect of temperature on the isotherms, while the Polynomial equation could. The mean adsorptive monolayer moisture content (*M*_0_) values in FOS and inulin samples were predicted as 7.29% and 7.94% wet basis, respectively. The heat of moisture sorption of FOS and inulin approached that of pure water at about 32.5% and 22.5% wet basis (w.b.) moisture content (MC), respectively. Fourier Transform Infrared Spectroscopy (FTIR) showed that the peaks in inulin with absorbance values above 0.52 and in FOS with absorbance values above 0.35 were at 1020, 1084, and 337 cm^−1^; these could represent the amorphous structure (primary alcohol C-OH), C-O group, and hydroxyl functional group, respectively. Microscopic structure analysis showed that inulin powder particles were more round-shaped and adhered together, resulting in hygroscopic and sticky characteristics, with a maximum equilibrium moisture content (EMC) of 34% w.b. In contrast, the FOS powders exhibited irregular amorphous particles and a maximum EMC of 60% w.b. As hydrogels, 3–10% FOS or inulin addition reduced the peak, trough, final, breakdown, and setback viscosities of rice starch pasting, but increased the peak time and pasting temperature. FOS addition gave stronger reduction in the setback viscosity and in amylose retrogradation of rice starch pasting than inulin addition. The differential scanning calorimeter (DSC) showed 3–10% FOS addition reduced the amylopectin aging of retrograded paste of rice starch, but 5–7% inulin addition tended to reduce. These results suggest that FOS and inulin have strong hygroscopic properties and can be used to maintain the freshness of starch-based foods. These data can be used for drying, storage, and functional food design of FOS and inulin products.

## 1. Introduction

Inulins are linear polysaccharides formed by fructose, usually with a glucose at the end; their degree of polymerization (DP) is usually 2–60 [1]. Fructooligosaccharides (FOSs) are a mixture of 1-kestose (GF2) to fructooligosaccharide (GF7) and fructodisaccharide (F2) to fructooligosaccharide (F8), produced from chicory (*Cichorium intybus*) or Jerusalem artichoke (*Helianthus tuberosus*) by partial enzymatic hydrolysis or membrane separation, purification, and drying, or a mixture of 1-kestose (GF2) to kestohexaose (GF5) produced from sucrose by the action of β-fructofuranosidase from *Aspergillus niger* or *Aspergillus oryzae* followed by purification and drying [2]. The functional food concept has recently become one of the essential elements to healthy nutrition and healthy living [3]. The utilization of FOSs and inulins in the food industry has grown in recent years because they offer the benefits of dietary fiber and can be employed as a carbohydrate or fat replacer due to their lower calorie production [4]. They also exhibit prebiotic functions, stimulating the growth of *Bifidobacterium* and *Lactobacillus* in the large intestine; thus, they can be employed in functional food formulations [5]. However, the equilibrium moisture content (EMC) of FOSs and inulins at similar physiological condition should be determined.

China has produced Jerusalem artichoke and chicory root crops since 2000. China’s inulin output was less than 1000 tons in 2009, but it surged to 15,000 tons by 2019 and exceeded 22,000 tons in 2023 [6]. However, amorphous inulins can exhibit caking and lumping phenomena during storage and transportation, and the reliable storage moisture content and optimal humidity conditions are required for the storage of inulin products.

During storage, the quality of food powders is influenced by ambient factors such as temperature, relative humidity, and oxygen level [7]. The water activity and moisture content are also considered parameters when describing food stability. With changes in the moisture content of inulin powder, various physical changes can occur, such as agglomeration, stickiness, and caking [8]. Moisture sorption isotherms shows that the equilibrium moisture content (EMC) of a food is related to its water activity at certain given temperatures [9]. Povolny et al. [8] investigated that the sorption isotherm behavior of commercial inulin samples is influenced by their degree of polymerization (DP) and molecular weight distribution. Ronkart et al. [10] reported the moisture adsorption and desorption isotherms of a kind of inulin powder (average DP of 10) supplied by Belgium Warcoing at a storage temperature of 20 °C. Zimeri and Kokini [11] measured the moisture sorption isotherm of inulin (Raftiline HP, DP ≥ 25) at 25 °C by applying the saturated salt solution and gravimetric method. Luo et al. [12] determined the 25 °C, 30 °C, and 45 °C adsorption isotherms of inulin with DP 10–12 using dilute sulfuric acidwater solution to condition the relative humidity and the static gravimetric method for determination, and revealed J-shaped isotherms. Jirayucharoensak et al. [5] used six saturated salt solutions to measure the 0 °C, 10 °C, and 30 °C adsorption isotherms of inulin powder. However, to date, there is a lack of studies of the adsorption/desorption isotherms and temperature-dependent EMC modeling of inulin and FOS powder products produced in China, plus limited linkage to starch retrogradation endpoints.

Thermodynamic functions like isosteric heat of sorption can be calculated from moisture sorption isotherm and have practical use in modelling energy consumption in the drying process of biomaterials [9]. No report on the isosteric heat moisture sorption of FOS and inulin products has been published to date. In order to overcome the difficulties associated with measuring the moisture adsorption and desorption isotherms of gel-like food powder samples, this study employed high-purity nitrogen as a drying gas and mould inhibitor, controlled the equilibrium relative humidity of the sample chamber with saturation vapour, and used the dynamic gravimetric method to measure the moisture adsorption and desorption curves of FOS and inulin powder samples, showing how FOS and inulin interacts with the moisture in the air. The aim was to predict the shelf life, determine the monolayer moisture content and proper storage conditions, give suitable drying and packaging design, and foretell the EMCs at physiological condition for gel-forming FOS and inulin products.

## 2. Results and Discussion

### 2.1. Experimental EMC/a_w_ Data for FOS and Inulin Samples

**Figure 1:**
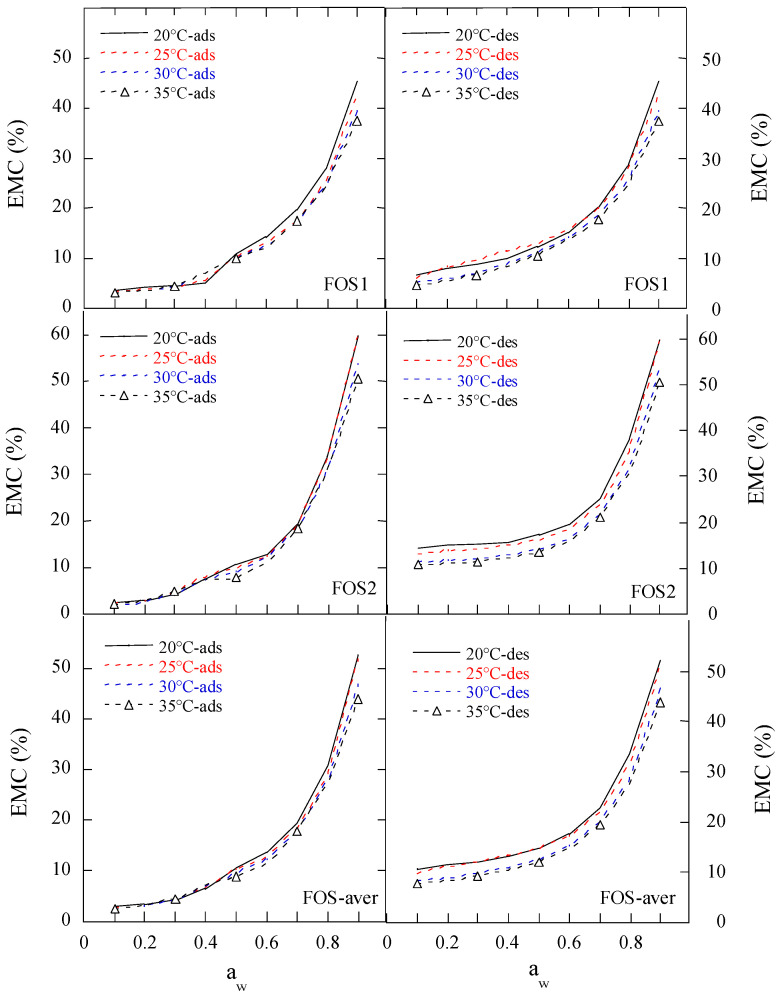
The measured moisture sorption isotherms of two FOS samples. Notes: ads, adsorption; des, desorption; FOS1 and FOS2 are two FOS samples; FOS-aver is the average adsorptive or desorptive EMC/a_w_ data of two FOS samples.

**Figure 2:**
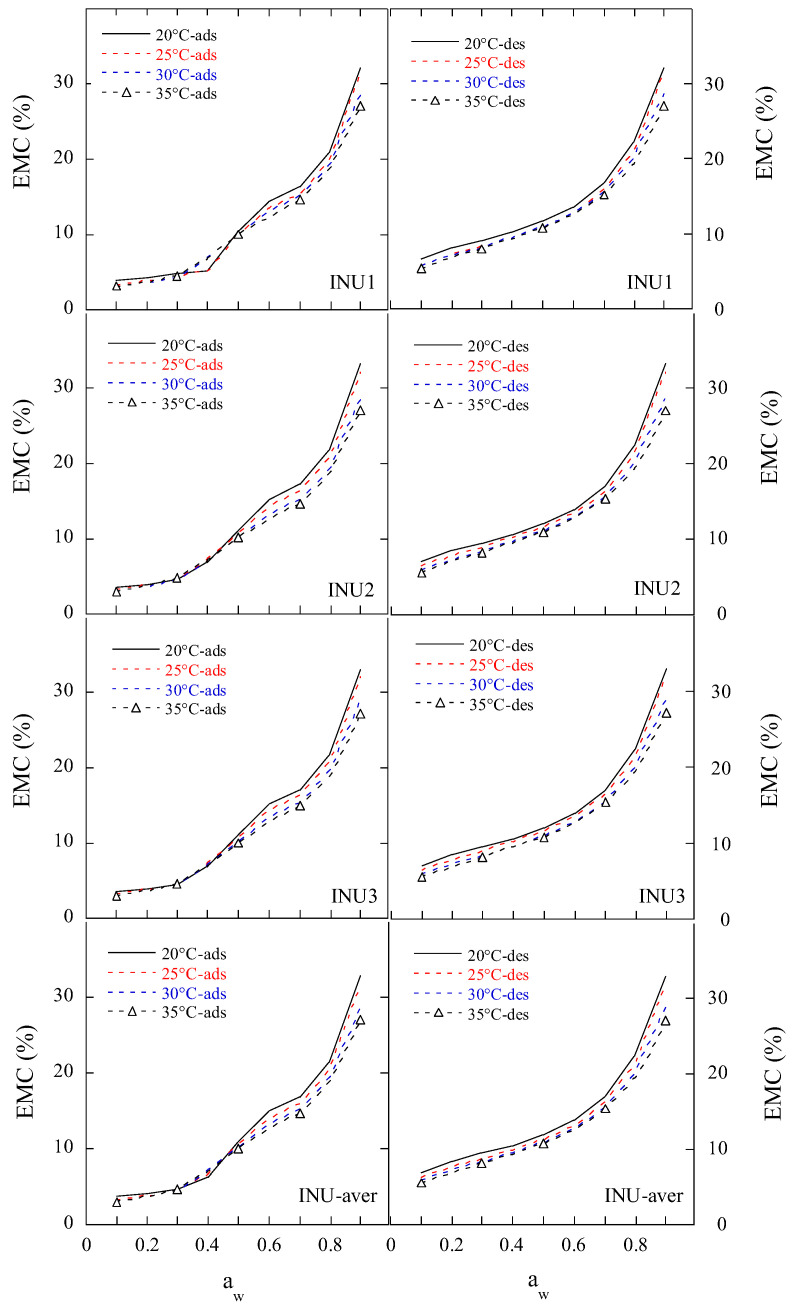
The measured moisture sorption isotherms of three inulin samples. Notes: ads, adsorption; des, desorption; INU1, INU2 and INU3 are three inulin samples; INU-aver is the average adsorptive or desorptive EMC/a_w_ data of three inulin samples.

The adsorption and desorption isotherms of the two FOS (FOS1 and FOS2) and three inulin (INU1, INU2, and INU3) samples (Table 1) were measured at four temperatures (20 °C, 25 °C, 30 °C, and 35 °C) and water activity ranging from 0.1 to 0.9, as shown in Figure 1 and Figure 2. Both the adsorption and desorption isotherms were sigmoidal in shape. The equilibrium moisture contents (EMCs) of FOS and inulin samples at constant a_w_ decreased as the sorption temperature increased, because the kinetic energy of water molecules is high and water adsorption is low at high temperatures. With increase in the mobility of water molecules, water molecules cannot bind to the samples through hydrogen bonds, thereby the moisture content (MC) of the samples decreased with an increase in temperature [13].

For the FOS and inulin samples, the adsorption isotherms exhibited type-II sigmoidal curves with at least two inflection points, but the desorption isotherms exhibited smooth type-II sigmoidal curves. At the same temperature, there were big differences between the desorption and adsorption isotherms in the range of 0.1 to 0.69 a_w_. With an increase in the initial moisture content (IMC) of the samples, the isotherms at the same temperature were raised slightly for desorption or adsorption. The EMC of the samples showed a very slow increase at 0.1–0.2 a_w_ and a slow increase at 0.21–0.6 a_w_ for four temperatures, indicating water was only adsorbed on the surface, and monolayer and multilayer water regions were successively formed, while the solubility of sugar components and moisture content increased at all temperatures as the a_w_ further increased [14].

At 0.3–0.7 a_w,_ there were significant inflection points in the adsorption isotherms for the two FOS and three inulin samples, indicating surface adsorption and bulk absorption of gel-forming polysaccharides.

At 0.1–0.9 a_w_ and 20–35 °C, the EMC range of the FOS samples was 2.0–59.5% and 4.8–59.5% wet basis (w.b.) for adsorption and desorption, respectively, while that of the inulin samples was 3.0–33.4% and 5.4–33.4% w.b. for adsorption and desorption, respectively. The EMC range of the inulin samples was similar to that of Jirayucharoensaket al. [5], where the EMCs of inulin powder were in the range of 2.1–36.8% at 0.1–0.9 a_w_ and 0–30 °C. Ronkart et al. [10] suggested that the sigmoidal-shaped curves obtained for both desorption and adsorption isotherms of industrial spray-dried inulin (average DP of 5) were indicative of type II isotherms, according to Brunauer’s classification. Figure 1 and Figure 2 show that the desorption isotherms of Chinese FOS and inulin samples were more like type IIa sigmoidal curves.

The large hysteresis between adsorption and desorption occurred at a_w_ < 0.7 for the FOS samples, and at a_w_ < 0.6 for the inulin samples, suggesting the similar hygroscopic behavior of FOS and inulin to that of polydextrose reported by Liu et al. [15].

### 2.2. Fitting of Moisture Sorption Equations to Experimental Sorption Data

The EMC equations in Table 2 were used to fit the measured EMC/a_w_ data of two FOS and three inulin samples. The statistical parameters such as residue sum of squares (*RSS*), standard error (*SE*), determination coefficient (*R*^2^), and relative percentage error (*MRE*) used to compare the model fits are given in Table 3, Table 4, Table 5, Table 6 and Table 7. The seven equations, namely Boquet, Ferro–Fontan, Guggenheim–Anderson–de Boer (GAB), Generalized D’Arcy and Watt (GDW), Peleg, the modified GAB (MGAB), and our developed seven-parameter Polynomial, all exhibited good fits to the measured sorption isotherm data of the FOS and inulin samples in the range of 0.1–0.9 a_w_, although GAB had larger *MRE* values for the adsorptive EMC data in the FOS1, FOS2, and INU2 samples, and Polynomial exhibited a larger *MRE* value for the adsorptive EMC data in the FOS2 sample.

The GAB and Peleg equations offered better fitting results to the desorption isotherms of the three inulin samples because the *MRE* values were <9.44% (Table 3 and Table 5). These results are similar to the results obtained for inulin powder containing FOS and inulin-type fructan at 0–30 °C and 0.1–0.9 *a*_w_ by Jirayucharoensak et al. [5].

The monolayer moisture content (*M*_0_) is considered as an ideal moisture content to avoid changing the product’s quality [3], which was estimated by the parameter a in GAB and MGAB models in the present study. The GAB equation indicated that the monolayer moisture contents of three inulins were 5.07–7.76% w.b. and 6.29–7.37% w.b for adsorption and desorption, respectively. These values are smaller than those (8.47–10.16% w.b.) of inulin powder at 0–10 °C measured by Jirayucharoensak et al. [5].

The average adsorption *M*_0_ values in GAB were 7.071 and 7.485% w.b. for FOS and inulin, respectively, and those in MGAB were 7.507 and 8.392% w.b. for FOS and inulin. Meanwhile, the average desorption *M*_0_ values in GAB were 6.182 and 6.271% w.b. for FOS and inulin, respectively, and those in MGAB were 6.273 and 6.383% w.b. for FOS and inulin. The adsorption *M*_0_ values in FOS and inulin were all below the maximum monolayer moisture content (9.1% w.b.) given by Gül et al. [3] and Labuza [19].

Compared with the other equations used in the present study, the modified Chung–Pfost (MCPE) in a form of $M=f\left(a_{w},t\right)$ fell short of expectation due to 0.875–0.960 of *R*^2^ and 20.76–79.00% of *MRE* for adsorptive samples, and 0.815–0.937 of *R*^2^ and 13.05–26.60% of *MRE* for desorptive samples (Table 7), but the MCPE in a form of $a_{w}=f\left(M,t\right)$ gave 0.931–0.970 of *R*^2^ and 15.73–22.41% of *MRE* for adsorptive samples, and 0.866–0.973 of *R*^2^ and 11.93–30.53% of *MRE* for desorptive samples; thus the MCPE parameters in FOS-aver and INU-aver samples could be used to analyze the isosteric heats of moisture sorption.

Further comparison of the sorption equations was performed for the five sets of FOS and inulin isotherm data (Table 8), and the average values of the *R*^2^ and error parameters (*RSS*, *SE*, and *MRE*) were calculated. For adsorption, the equations in a form of $M=f\left(a_{w},t\right)$ were ranked in the following order of accuracy from the highest to the lowest: Ferro–Fontan, GDW, Boquet, Peleg, MGAB, Polynomial, GAB, and MCPE. In the case of desorption behavior, the order was Polynomial, MGAB, Peleg, Boquet, GDW, Ferro–Fontan, GAB, and MCPE. For adsorption, the Ferro–Fontan and GDW equations offered good descriptions of the equilibrium moisture data of the five samples of FOS and inulin in the range of 0.1–0.9 a_w_, and for desorption, the Polynomial and MGAB equations were the best fitting. The coefficients of the best-fitting equations for the average EMC/a_w_ data in the FOS and inulin samples are summarized in Table 3, Table 4, Table 5 and Table 6. These calculated coefficients can be used to describe the dehydration process of FOS or inulin and can be employed to improve the physical control of moisture content during packaging and storage.

### 2.3. Prediction of Moisture Desorption and Adsorption Isotherms by the Best-Fitting Equation

**Figure 3:**
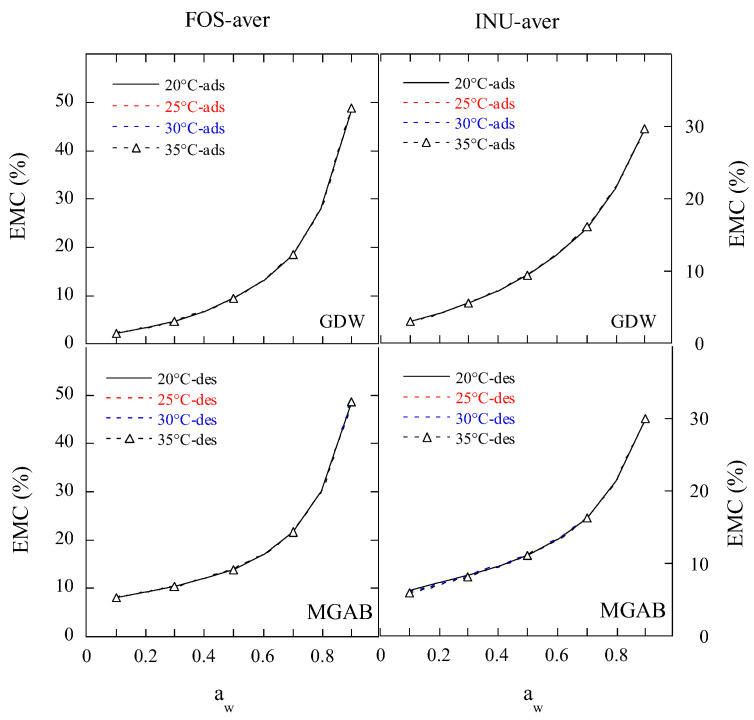
The predicted mean isotherms of FOS or inulin samples by GDW and MGAB equations. Note: FOS-aver is the mean adsorptive or desorptive EMC/*a*_w_ data of two FOS samples; INU-aver is the mean adsorptive or desorptive EMC/*a*_w_ data of three inulin samples; ads, desorption; des, desorption; Generalized D’Arcy and Watt; MGAB, the modified GAB.

**Figure 4:**
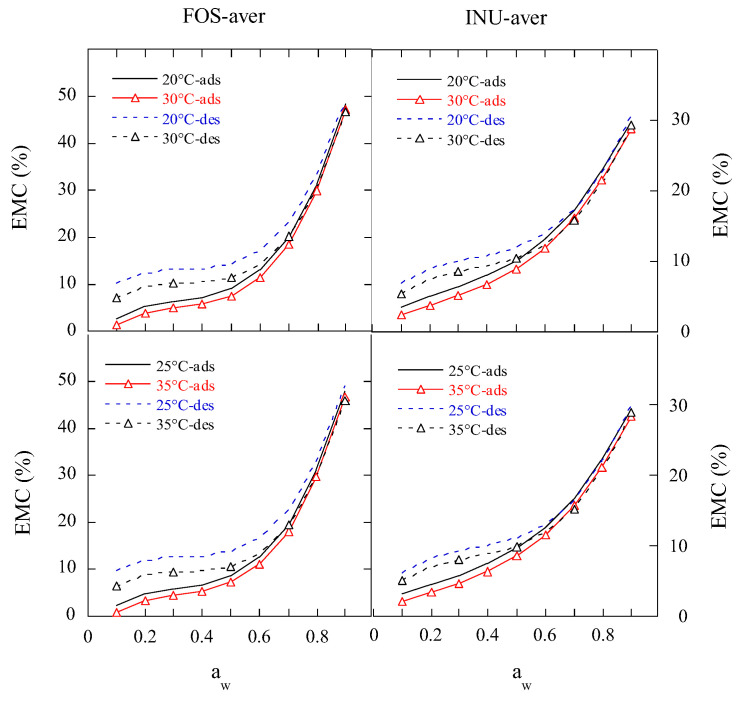
The predicted mean isotherms of FOS or inulin samples by Polynomial equation. Notes: FOS-aver is the mean adsorptive or desorptive EMC/*a*_w_ data of two FOS samples; INU-aver is the mean adsorptive or desorptive EMC/*a*_w_ data of three inulin samples; ads, adsorption; des, desorption.

The predicted mean adsorption and desorption isotherms of the FOS and inulin samples at 20 °C, 25 °C, 30 °C, and 35 °C using GDW and MGAB are displayed in Figure 3. It can be seen that these isotherms exhibited smooth curves and did not show the effect of temperature. Figure 4 presents the predicted mean isotherms of the FOS and inulin samples by the Polynomial equation. The Polynomial equation displayed the effect of temperature. At *a*_w_ ≤ 0.8, the hysteresis between adsorption and desorption became bigger with decreases in the *a*_w_ at the four temperatures. At a_w_ ≤ 0.6, the adsorption and desorption isotherms of the FOS sample were lower than those of the inulin sample at the same temperature. These results suggest that the difference might occur due to the monolayer and multilayer water molecules sorption properties of the FOS and inulin samples.

**Figure 5:**
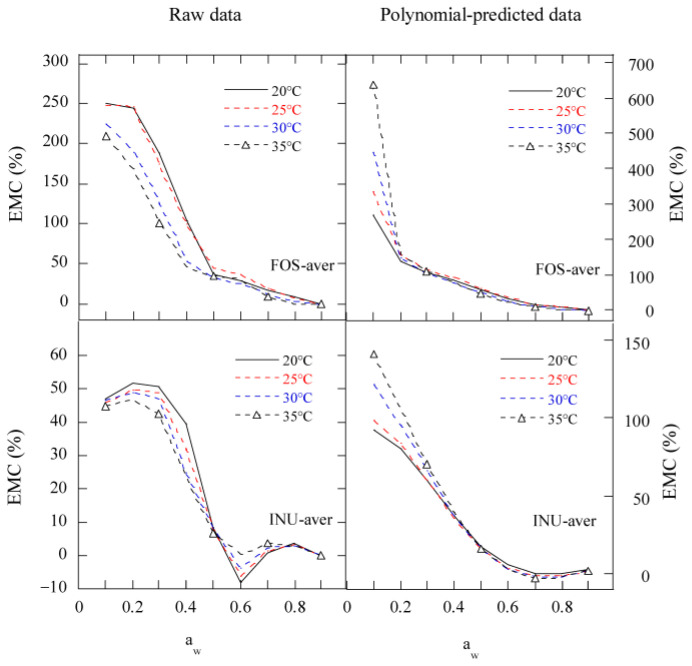
The hysteresis degree of inulin and FOS. Notes: FOS-aver is the mean adsorptive or desorptive EMC/*a*_w_ data of two FOS samples; INU-aver is the mean adsorptive or desorptive EMC/*a*_w_ data of three inulin samples.

The raw hysteresis degree curve of moisture sorption in FOS decreased so sharply with increases in the water activity below 0.5 *a*_w_, and then decreased gradually with increasing water activity. With increases in temperature, the hysteresis degree curve of FOS moved down (Figure 5). The fitted hysteresis degree curves of FOS using the Polynomial equation could be divided into three stages at ≤0.2 *a*_w_, 0.21–0.7 *a*_w_, and 0.71–0.9 *a*_w_, corresponding to a fast decrease in the hysteresis degree, a slow decrease, and a flat line, respectively. With decreases in the temperature, the Polynomial fitted hysteresis degree curve of FOS at 0.1–0.2 *a*_w_ moved down. These results suggest that FOS has three inflection points at *a*_w_ = 0.2, 0.5, and 0.7, respectively.

The raw hysteresis degree curve of moisture sorption in inulin first increased at 0.1 to 0.2 *a*_w_, then decreased sharply at 0.3 to 0.6 *a*_w_, but increased at 0.6 to 0.8 *a*_w_, and finally decreased slowly to 0.9 *a*_w_. With increases in temperature, the hysteresis degree curve of inulin moved down (Figure 5). The fitted hysteresis degree curves of inulin using the Polynomial equation could be divided into three stages at <0.2 *a*_w_, 0.2–0.6 *a*_w_, and 0.61–0.9 *a*_w_, corresponding to a fast decrease, slow decrease, and flat line in the hysteresis degree, respectively. With decreasing temperature, the Polynomial fitted hysteresis degree curve of inulin at 0.1–0.2 *a*_w_ moved down. These results suggest that inulin has at least five inflection points at *a*_w_ = 0.2, 0.3, 0.4, 0.6, and 0.7, respectively.

At 0.1 *a*_w_, the large hysteresis between the desorption and adsorption isotherms might derive from the reason that the semi-crystalline inulins or FOS are more hygroscopic than their amorphous counterparts. The further work will measure the glass transition temperature (*T*_g_) and crystallinity of inulin or FOS samples by DSC and XRD to corroborate this semi-crystalline transition hypothesis.

### 2.4. The Isosteric Heat of Sorption of FOS and Inulin Samples

**Figure 6:**
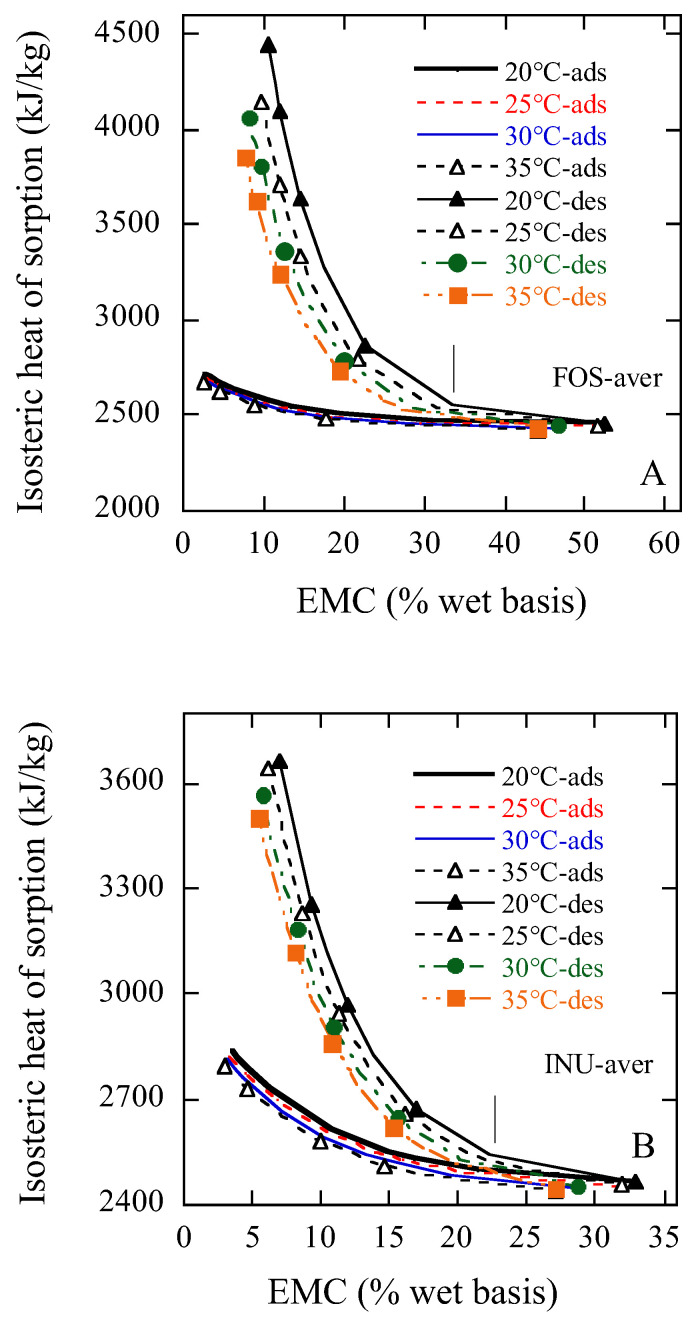
Comparison of adsorption and desorption isosteric heats of FOS and inulin at different temperatures predicted by the Modified Chung–Pfost equation. Notes: (**A**), FOS-aver; (**B**), INU-aver.

The coefficients a, b, and c of the MCPE equation with a form of $a_{w}=f\left(M,\;t\right)$ were used to calculate the sorption isosteric heats. Figure 6 shows the effect of moisture content (MC) on adsorption and desorption isosteric heats of FOS and inulin. The isosteric heats for both desorption and adsorption of FOS (Figure 6A) decreased rapidly with the increase in the sample MC until a moisture content of 32.5% w.b. (shown by short line) was achieved, but above 32.5% they decreased smoothly with increasing moisture content. At lower MCs below 30%, the isosteric heats of both FOS desorption and adsorption at lower temperatures were higher than those at higher temperatures. The isosteric heats of FOS desorption were higher than those of adsorption below 40% MC, but thereafter there was a minor difference found between desorption and adsorption (Figure 6A). The isosteric heats for both desorption and adsorption of inulin (Figure 6B) decreased rapidly with the increase in the sample MC until a moisture content of 22.5% w.b. (shown by short line) was achieved, but above 22.5% they decreased steadily with increase in MC.

The rapid rise in the heat of sorption at low MC might be due to the existence of considerably active polar sites on the surface of FOS and inulin samples, which were covered with water molecules forming a mono-molecular layer [9]. The reduction in the isosteric heats with higher amounts of sorbed water could be quantitatively explained by taking into account that sorption initially develops on the most active available sites bringing about high interaction energy. When these sites are occupied, sorption occurs on the less active ones, giving rise to lower heats of sorption [9]. In low MCs, the values of the isosteric heats were higher than the latent heat of water vaporization, showing that the binding energy of the water molecules and the sorption sites was higher than the energy which keeps the pure water molecules together in the liquid phase [20]. At high MCs, there was no appreciable difference between the sorption isosteric heat and the latent heat of water vaporization over the broad range of MCs. In the present study, the heat of sorption of FOS and inulin might approach that of pure water at about 32.5% and 22.5% w.b. MC, respectively. These isosteric heats can be used in drying calculation and modelling energy consumption in the drying process of FOS and inulin products.

### 2.5. FTIR Analysis of FOS and Inulin Samples

**Figure 7:**
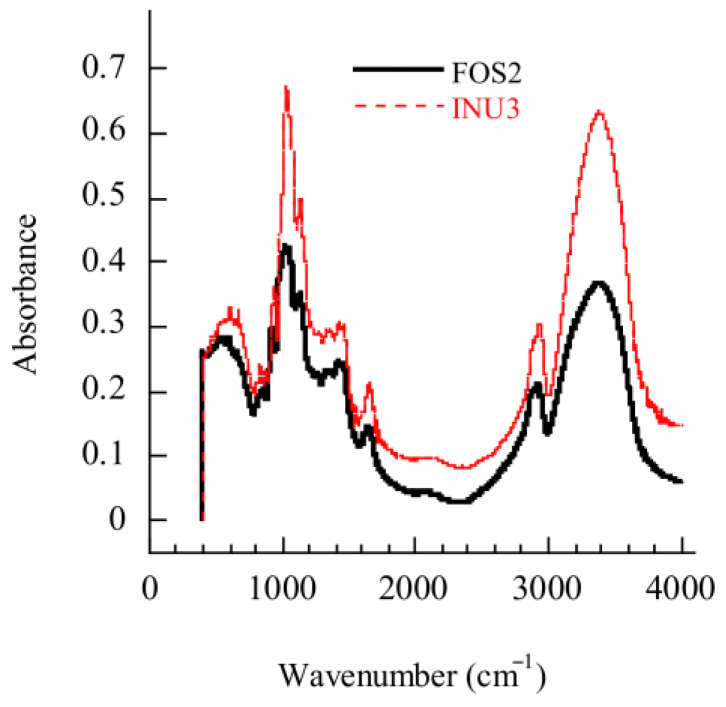
The absorbance of main peaks in the FTIR of FOS (FOS2) and inulin (INU3).

FOS powder had 11absorbance peaks in the FTIR spectrum, and inulin powder had 10 peaks (Figure 7 and Table 9). The peaks in inulin with absorbance above 0.52 and in FOS with absorbance above 0.35 were at 1020, 1084, and 3337 cm^−1^, which represent the amorphous structure (primary alcohol C-OH), C-O group, and hydroxyl functional group, respectively [21]. Peaks at 2892 and 2915 cm^−1^ show CH stretching and CH_2_ stretching, respectively [22]. Peaks at 1232, 1329, 1405, and 1628 cm^−1^ indicate C-OH and CH_2_OH, CH_3_ group, CH_2_ stretching and bending, and OH bending, respectively [23]. The peak at 925 cm^−1^ shows C-C stretching and C-O-C vibrations [24]. The peaks at 514, 556, and 814 cm^−1^ reflect skeletal vibrations of the fructose and glucose pyranose rings [25]. The peak at 749 cm^−1^ indicates C-H groups [22]. The main FTIR peaks in the present study are similar to the results of Pourfarzad et al. [22] who identified 14 FTIR peaks (577, 805, 875, 895, 1022, 1079, 1161, 1241, 1413, 1654, 2164, 2380, 2929, 3433 cm^−1^) in Serish inulin gel with a DP of 13.

### 2.6. Microstructure of FOS and Inulin Samples

**Figure 8:**
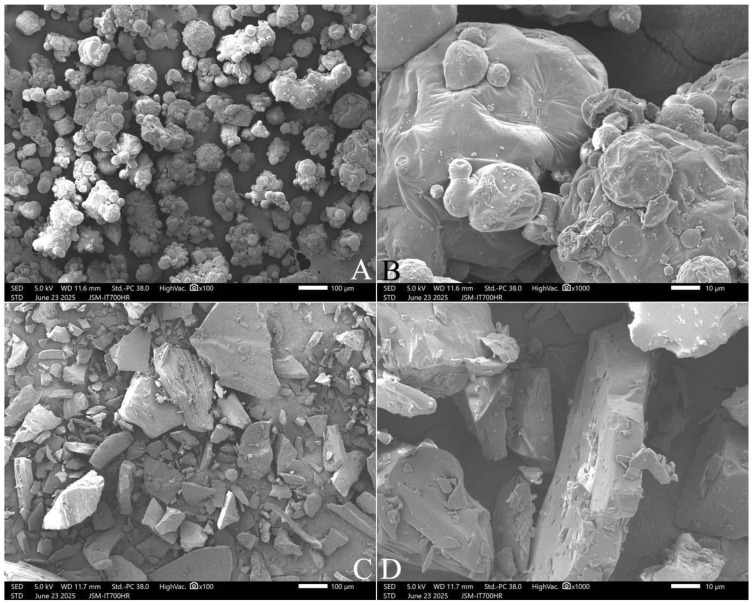
The microstructure of inulin (INU3) and FOS (FOS2). Note: (**A**,**B**), INU3; (**C**,**D**), FOS2. The photos were enlarged at 100× (**A**,**C**) and 1000× (**B**,**D**), respectively.

Figure 8 shows the microstructures of the inulin and FOS powders. The inulin powders were more round-shaped and adhered together. The FOS powders had irregular particles. The round-shaped and sticky microstructures of inulin reflect the hygroscopic and sticky characteristics of these powder samples. Inulin powders are sticky and adhere together at temperatures higher than the glass transition temperature [5,26]. The further work will determine the particle size distributions from automated image analysis to support statements about “round” versus “irregular” particles, and quantify shape descriptors and agglomerate size.

### 2.7. Effect of FOS and Inulin on the Pasting Parameters of Rice Starch

Table 10 shows the influence of adding FOS or inulin on the pasting parameters of rice starch. The 3–10% FOS or inulin addition reduced the trough, peak, final, breakdown, and setback viscosities of rice starch pasting, but increased the peak time and pasting temperature. The present results are similar to the decrease in peak, trough, breakdown, final, and setback viscosities of wheat flour pasting and an increase in pasting temperature with additional amounts of short-chain and long-chain inulins from 0 to 8% [27].

The peak viscosity revealed the expansion degree of a starch granule and the capability to bind water molecules in the warming process of starch; when the additional amount of FOS and inulin was 10%, the peak viscosityreduced by 25.9% and 29.6%, respectively. The significant decrease in peak viscosity might come from the better moisture absorption properties of FOS and inulin, and the higher hygroscopicity in FOS and inulin could inhibit water molecules from entering the amorphous region of starch granules, influencing starch gelatinization [28]. The breakdown viscosity mainly revealed the stability of rice starch paste in the heating process; the 10% addition of FOS and inulin reduced the breakdown viscosity by 45.8% and 31.0%, respectively, indicating FOS had a better effect on improving the stability of starch granules than inulin, probably due to the better water-holding ability at high temperature [29]. The maximum increase inthe pasting temperature at the 10% addition amount suggested that the incorporation of FOS and inulin produced the FOS-starch or inulin-starch mixture more challenging to gelatinize.

The setback viscosity could reveal the recrystallization degree of starch during the cooling course of starch gelatinization, especially in the recrystallization and rearrangement of amylase. The 10% addition of FOS and inulin in rice starch reduced the setback viscosity by 22.9% and 16.4%, respectively. FOS and inulin could compete with rice starch for water molecules during starch gelatinization, and the hydrogen bonding of starch molecules was hindered by the interaction of FOS and starch molecules or inulin and starch molecules, making it more difficult for amylase to form a double-helix structure [30]. FOS addition produced a stronger reduction in the setback viscosity of rice starch pasting than inulin addition, indicating that these two polymers delay amylose retrogradation and could be used to improve the freshness of cooked rice as gel-forming agents.

The magnitude of setback viscosity measured by a rapid viscosity analyzer (RVA) is considered to reveal the retrogradation tendency of amylase in a starch paste, while in the case of retrograded starch, the endotherm of a differential scanning calorimeter (DSC) gives quantitative measure of enthalpy change and transition temperatures for the melting in recrystallized amylopectin [31]. The present study determined the aging of retrograded rice starch paste stored at 4 °C after 21 days. Table 11 shows the influence of adding FOS or inulin on the thermal parameters of rice starch. Compared with the control sample, 3–10% FOS addition increased the gelatinization enthalpy of rice starch (Δ*H*), and 3–7% FOS addition reduced the peak temperature of gelatinization (*T*_p_), while 5–7% inulin addition kept the Δ*H*, and 3–10% inulin addition kept the *T*_p_. The 3–10% FOS and inulin addition both kept the conclusion temperature of gelatinization (*T*_c_). Furthermore, 3–10% FOS addition reduced the amylopectin aging of retrograded paste, but 5–7% inulin addition tended to reduce.

Tudoricâ et al. [32] showed that compared with the control sample, 7.5–10% inulin addition increased the peak and conclusion temperatures of gelatinization in raw pasta but decrease the gelatinization enthalpy value. In the present study, 7–10% inulin addition in rice starch kept the peak and conclusion temperature of gelatinization, which is different tothe reduction in starch gelatinization events in raw pasta by 7.5–10% inulin addition. These results suggest that rice starch and raw paste have different gelatinization responses to inulin addition.

General Linear Model (GLM) analysis (Table 12) further showed that, compared with the control sample, FOS addition increased the Δ*H*, but decreased the *T*_p_ and the aging of retrograded paste, while inulin addition kept the Δ*H* and the aging of retrograded paste, and increased the *T*_p_. Both FOS and inulin addition kept the *T*_c_ value. With the increase in the addition amount of two prebiotics, the *T*_p_ increased, and the aging of retrograded paste decreased. The 5–7% addition amount increased the Δ*H*, and the 3–5% addition amount decreased the *T*_c_ value.

These results suggest 3–10% addition significantly reduced the aging of retrograded paste of rice starch, but 5–10% inulin addition also tended to reduce it. Compared with inulin, FOS has stronger competitiveness with rice starch for water molecules and thus delays starch retrogradation.

Inulin is currently more widely used as a food ingredient; to avoid its caking and lumping complaints, it is in urgent to know how it interacts with the moisture in air. Few studies have investigated its moisture sorption properties, possibly because there is a need for strict control of temperature and humidity over a long duration in order to determine its desorption isotherms. The present study is the first to measure the adsorption and desorption isotherms of inulin samples at 0.1–0.9 *a*_w_ over a 45-day period. At 0.9 *a*_w_, the three inulin samples exhibited desorption EMCs of 27.0–33.36% w.b.at 20–35 °C. Inulin has a higher water-binding capacity than cereal grain (20–25% w.b., [9]), with a water-binding capacity of about 2:1 [33]. It is generally considered that amorphous materials are more hygroscopic than crystalline ones, but Ronkart et al. [34] observed that under low humidity (0–12%), semi-crystalline inulins are more hygroscopic than their amorphous counterparts. This might explain the large hysteresis between inulin adsorption and desorption at 0.1 *a*_w_, because the adsorption inulin powder is in an amorphous state, but the desorption inulin powder is in a semi-crystalline state.

According to the classification of sorption isotherms by Blahovec and Yanniotis [35], the shape of moisture sorption isotherms of FOS and inulin samples in the present study can be classified as type IIa isotherm curves. Gül et al. [3] categorized the moisture sorption isotherms of agglomerated boza powder as type III isotherms; we regarded that they should be type IIa isotherm curves because type III isotherms represent crystalline solids such as sugars and salt, and crystalline lactose powder adsorbs very little water over the low *a*_w_ range (0–0.85), but adsorbs significant amounts of water at a_w_ above 0.85 [36].

In the present study, the FOS (DP 2–8) and inulin (DP 2–60) powders exhibited type IIa isotherm curves, closer to that of Raftilose P95 (DP 5) [8], rather than the type IIb isotherm of Raftiline HP (DP 23) from Orifti, Tienen Belgium [8]. Compared with a type IIb isotherm, type IIa isotherm curves have lower moisture contents at *a*_w_ below 0.6 and higher moisture contents at higher *a*_w_ [8,35]. According to the result of Mazza [37], the water insoluble fractions in Jerusalem artichoke flour had a type II isotherm curve. Thus, we conclude that the FOS (DP 2–8) and inulin (DP 2–60) powders in the present study are water soluble fractions, and the short chain lengths of FOS and inulin have more hydroxyl groups available for water molecules to bind to at higher water activity during sorption.

Jirayucharoensak et al. [5] calculated the suitable water activity conditions for the storage of inulin samples using the Lewicki-3 model and found that the moisture content of inulin powder should be kept at ≤5.75% w.b. during storage and the ambient relative humidity should not exceed 18.32% and 37.12%, respectively. In the present study, when the moisture content was 5.75% w.b., the adsorption ERHs of inulin at 10, 20, and 30 °C calculated using the GAB equation were 17.99%, 26.58%, and 32.39%, respectively, which is very close to the results of Jirayucharoensak et al. [5]. The adsorption ERHs of the FOS samples at 10 °C, 20 °C, and 30 °C were 23.01%, 30.32%, and 35.07%, respectively, using the GAB equation. These results suggest that inulin and FOS powder should be kept in packaging that can prevent moisture transfer from the surrounding air into the package, especially in subtropical regions such as South China, where the relative humidity of the environmental air is usually high.

Our previous report [15] showed five polydextrose samples at approximate physiological conditions like 35 °C and 0.9 *a*_w_ had the EMCs of 46.4–48.5%. In the present study, two FOS samples and three inulin samples at 35 °C and 0.9 *a*_w_, respectively, had the EMCs of 37.5–50.5% and 27.0–27.3%. Polydextrose, FOS, and inulin have a respective average DP of 12, 2.7–4, and 10 [15,38]. These combinations of average DP and the EMC value at physiological conditions might decide their functions that polydexrose plays a role based on dietary fiber, FOS makes a contribution by high-efficiency prebiotics and metabolic regulation, and inulins act as prebiotics and multi-effect regulation [38,39]. Further research will need to define the effects of FOS and inulin on the rice cooking properties, rice textural profile, and the microstructure of cooked rice, as well as the thermal, pasting, and thermo-mechanical properties of rice flour, and determine the optimal levels of FOS or inulin addition.

## 3. Conclusions

The present study is the first to determine the adsorption and desorption isotherms of FOS and inulin samples at 0.1–0.9 a_w_ over a 45-day period, and obtain the isotherms of 20–35 °C. Their shape showed type IIa isotherm curves. At 0.1 a_w_, the large hysteresis between the desorption and adsorption isotherms might come from the reason that the semi-crystalline inulins or FOSs are more hygroscopic than their amorphous counterparts. For adsorption, the Ferro–Fontan and GDW equations are best for describing the isotherms of FOS and inulin, respectively, and for desorption, the Polynomial and MGAB equations are best. Inulin powder is more round-shaped and the particles adhere together, resulting in hygroscopic and sticky characteristics, with a maximum EMC of 34% wet basis (w.b.). In contrast, FOS powders are characterized by irregular amorphous particles with a maximum EMC of 60% w.b. The mean adsorptive monolayer moisture content (*M*_0_) values in FOS and inulin samples were predicted as 7.289% and 7.939% wet basis, respectively. The heat of sorption of FOS and inulin approaches that of pure water at about 32.5% and 22.5% w.b. MC, respectively. Both FOS and inulin were found to have rich amorphous structures (primary alcohol C-OH), C-O groups, and hydroxyl functional groups. Two FOS samples and three inulin samples at approximate physiological conditions like 35 °C and 0.9 a_w_, respectively, had the EMCs of 37.5–50.5% and 27.0–27.3% w.b. These powders can be used as gel-forming agents to maintain the freshness of starch-based foods by competing with starch for water molecules during pasting. This study provides useful insights for the dehydration, storage, packaging, and food addition of FOS and inulin products. The effects of FOS and inulin addition on the rice cooking properties, textural profile, thermal properties, and the microstructure of cooked rice will be studied.

## 4. Materials and Methods

### 4.1. The Samples

Two samples of FOS and three inulins were provided by Runloy Biotechnology Co., Ltd., Shanghai, China (Table 1). The two FOS powders met the China national standard GB1903.40-2022 with DP 2–8 [2]. The three inulin samples met the quality requirement of GB/T 41377-2022 with DP 2–60 [1]. The initial moisture contents (MCs) of the samples were determined by the AOAC method [40].

### 4.2. Moisture Desorption and Adsorption Isotherms and Their Fitting

The moisture desorption and adsorption isotherms of the FOS and inulin samples were measured with a dynamic moisture sorption analyzer (SPS11-10μ, ProUmid GmbH & Co. KG, Ulm, Germany), as described by Liu et al. [15]. This instrument is a merged system for self-acting gravimetric determination of the water vapor adsorption and desorption of eleven samples in a test atmosphere chamber with controlled water activity (a_w_) and temperature. The temperature accuracy is ±0.1 K and the precision of water activity varying with time is ±0.6% at (23 ± 5 °C) in the range of 0–1 a_w_. The equilibrium moisture contents (EMCs) of two parallel samples (each *ca*. 2.0000 g) under one of four constant temperatures (20 °C, 25 °C, 30 °C, and 35 °C) over an a_w_ range of 0.1–0.9 were determined with the use of deionized water to produce humidity and high-purity nitrogen to blow the samples dry and prevent them from going mouldy. The interval size between gravimetric cycles was set at 10 min. The adsorption measurement cycle was begun at 0.1 a_w_ and first boosted with a 0.1 a_w_ step to 0.2 a_w_. Then, a_w_ was boosted successively to 0.3, 0.4, 0.5, 0.6, 0.7, 0.8, and 0.9. The desorption measurement cycle was carried out from 0.8 a_w_ to 0.1 a_w_ with 0.1 a_w_ step and seven steps. The time per cycle was set up to a minimum of 50.1 min and a maximum of 50.1 h. The default weight limit was +100%, and balance bandwidth (dm/dt) was ±0.01%/40 min. During a measurement cycle, the samples were automatically placed on an analytical balance (0.00001 g) and weighed. The sample pan remained unloaded and was adoptedfor drift compensation of the measured values. The recorded data were analyzed using SPS-Toolbox Basic Rel. 1.15 software.

The experimental EMC/a_w_ data were used to draw isotherms in Kaleidagraph version 4.54 software [41], with the a_w_ and EMC data shown on the *x*- and *y*-axis, respectively. The EMC equations in Table 2 were employed to fit the actually measured moisture isotherms of the samples.

To show the influence of temperature on the moisture isotherms, we supposed that EMC is the function of temperature and a_w_, and developed a seven-parameter Polynomial,

(1) $$M=a+b\cdot t+c\cdot t^{2}+d\cdot t^{3}+e\cdot a_{w}+f\cdot a_{w}^{2}+g\cdot a_{w}^{3},$$

where *M* is equilibrium moisture content (%w.b.), *t* is temperature (°C), *a*_w_ is water activity (decimal), and a–g are parameters.

Fitting was carried out by non-linear regression analysis in SPSS v17.1 for Windows (SPSS Inc., Chicago, IL, USA, [42]). The criteria used to decide the equation for the EMC/a_w_ data were the residue sum of squares (*RSS*), and standard error (*SE*), determination coefficient (*R*^2^), as well as the mean relative percentage error (*MRE*). Equations (2)–(5) were, respectively, used to calculate *RSS*, *SE*, *R*^2^, and *MRE*.

(2) $$RSS=\sum_{i=1}^{n}{\left(m_{i}-m_{\mathrm{pi}}\right)}^{2}$$

(3) $$SE=\sqrt{\sum_{i=1}^{n}{\left(m_{i}-m_{\mathrm{pi}}\right)}^{2}/\left(n-1\right)}$$

(4) $$R^{2}=1-\sum_{i=1}^{n}{\left(m_{i}-m_{\mathrm{pi}}\right)}^{2}/\sum_{i=1}^{n}{\left(m_{i}-m_{\mathrm{mi}}\right)}^{2}$$

(5) $$MRE\%=\frac{100}{n}\overset{n}{\underset{i=1}{\sum }}\left|\frac{m_{i}-m_{\mathrm{pi}}}{m_{i}}\right|$$

where *m*_i_, the experimental value; *m*_pi_, the predicted value; *m*_mi_, the average of experimental values; *n*, the total number of observations. The fitting of an equation to the EMC/*a*_w_ data of a sample was considered more satisfactory if the *MRE* value was lower than 20% [15].

The hysteresis degree (Hy) between moisture desorptionand adsorption was determined as,

(6) $$\mathrm{Hy}\;\left(\%\right)=\frac{EMC_{\mathrm{des}}-EMC_{\mathrm{ads}}}{EMC_{\mathrm{ads}}}\times 100$$

### 4.3. Determination of the Isosteric Heat of Sorption

The isosteric heat of moisture sorption (*h_s_*) is the quantity of energy needed to change one unit mass of product from liquid to vapour at a certain temperature and a_w_ [9]. The *h_s_* for FOS and inulin samples was assayed using the following equations [9]:

(7) $$\frac{h_{s}}{h_{v}}=1+\frac{P_{s}}{ERH}\cdot \frac{dt}{dP_{s}}\cdot {\left.\frac{\partial a_{w}}{\partial t}\right|}_{M}$$

(8) $$h_{v}=2501.33-2.363t$$

(9) $$P_{s}=\frac{6\times 10^{25}}{{\left(273.15+t\right)}^{5}}\cdot \exp\left(-\frac{6800}{t+273.15}\right)$$

(10) $$\frac{dP_{s}}{dt}=\frac{P_{s}}{\left(t+273.15\right)}\cdot \left(\frac{6800}{t+273.15}-5\right)$$

(11) $${\left.\frac{\partial a_{w}}{\partial t}\right|}_{M}=\frac{a\cdot a_{w}}{{\left(T+b\right)}^{2}}\cdot \exp\left(-c\cdot M\right)$$

where *h_s_* is the isosteric heat of moisture sorption (kJ/kg), *h_v_* is the latent heat of free water vaporization (kJ/kg), *t* is temperature, *M* is equilibrium moisture content (% wet basis), and $P_{s}$ is the saturated vapor pressure (Pa). Equation (7) was adopted to calculate the *h_s_*-to-*h_v_* ratio from $dP_{s}/dt$ and ${\left.\frac{\partial a_{w}}{\partial t}\right|}_{M}$, which can be calculated using Equations (10) and (11), respectively. The *h_v_* in Equation (8) is dependent on temperature. The *P_s_* was calculated using Equation (9). The ${\left.\frac{\partial a_{w}}{\partial t}\right|}_{M}$ term depends on the sorption isotherm equation used; this studyused the modified Chung–Pfost equation (MCPE), a, b, and c are the parameters of MPCE in the form of $a_{w}=f\left(M,\;t\right)$.

### 4.4. Fourier Transform Infrared Spectroscopy (FTIR)

The FTIR spectra of the FOS and inulin samples were measured on a Nicolet 6700 FTIR (Thermo Fisher Scientific, Greater Mumbai, MA, USA). The determination conditions were given as follows:64 scans, spectral resolution of 4 cm^−1^ with 100% T-line signal/noise ratio in the range of 4300–4400 cm^−1^. All measurements were treated with OMNIC software 9.1 (Thermo Fisher Scientific, Greater Mumbai, MA, USA). Samples were ground with potassium bromide (KBr) under a mass ratio of 100:1 and made into tablets. The scanning wavenumber was 400–4000 cm^−1^.

### 4.5. Scanning Electron Microscopy (SEM)

The FOS and inulin samples were separately fixed on a sample holder and then splashed with gold in the vacuum ion particle sprayer (JEC-3000FC, Japan Electronics Co., Ltd., Tokyo, Japan). The sputtering situation was given as follows: working distance of 10 mm, sputtering working pressure of 2.0 Pa, sputtering current of 30 mA, and sputtering time of 130 s. The samples were then set on the holder of the scanning electron microscope (JSM-IT 700HR, Japan Electronics Co., Ltd.), and photographed at the accelerating voltage of 25 kV with 100 to 1000 times magnification. The pressure in the observation room was 7.50 × 10^−8^ Pa, with the distance between the sample and the lens of 10mm and an emission current of 88 μA.

### 4.6. The Pasting Properties of Rice Starch with AddingFOS or Inulin

FOS (FOS2) or inulin (INU3) were added as gel-forming agents to rice starch powder (sigma) with *w*/*w* proportions of 0%, 3%, 5%, 7%, and 10%. The rapid viscosity analyzer (RVA–TecMaster, PertenRuihua Scientific Instrument Co., Ltd., Beijing, China) was adopted to measure the pasting parameters of the starch samples, according to the China national standard GB/T24852–2010 [43]. During each measurement, the stirring paddle was set up at 960 r/min for the initial 10 s, then reduced to 160 r/min within 20 s and remained at 180 r/min. The temperature was set initially at 50 °C for 1 min, then increased to 95 °C within 3.70 min and kept there for 2.5 min, before being lowered to 50 °C within 2.8 min and remained for 2 min.

### 4.7. Thermal Properties

FOS (FOS2) or inulin (INU3) was uniformly mixed with rice starch on amass basis to obtain adding levels of 0% to 10%. A 5.0 mg sample was used to measure the gelatinization parametersusingadifferential scanning calorimeter (DSC 214, NetzschGmbH, Selb, Germany), using the means of Wang et al. [44]. After measurement, the retrograded sample was placed in a numbered small plastic bag at 4 °C for 21 d and again determined for gelatinization enthalpy. The result wasanalyzedwith the same software. The aging of the retrograded starch paste was obtained using Equation (12):

Aging (%) = (Gelatinization enthalpy determined at day 21)/(Gelatinization enthalpy determined at day 0) × 100(12)

### 4.8. Data Analysis

Aside from parallel samples for the measurement of the EMC/*a*_w_ data, three replicates were measured to determine the physicochemical parameters of each FOS or inulin sample. SPSS software (Version 17.0, SPSS Inc., Chicago, IL, USA [42]) was adopted for data analysis. One-way analysis of variance and Duncan’s new multiple-range test were selected to compare pairs of means and multiple means, respectively. For considering the effect of FOS and INU species and addition amount, General Linear Model (GLM) analysis was adopted. Statistical significance is declared at *p* < 0.05.

## Figures and Tables

**Table 1:** The samples used by this study.

Sample No.	Species	Abbr.	Moisture Content (% Wet Basis)
a1	Fructooligosaccharide	FOS1	4.07
a2	Fructooligosaccharide	FOS2	2.49
a3	Inulin	INU1	4.25
a4	Inulin	INU2	3.89
a5	Inulin	INU3	3.87

**Table 2:** EMC equations used in the present study.

Equations	Formula	Reference
Ferro–Fontan	$M={\left[\frac{b}{\ln\left(\frac{a}{a_{w}}\right)}\right]}^{1/c}$	Saberi et al. [16]
GAB	$M=\frac{a\cdot b\cdot c\cdot a_{w}}{\left(1-b\cdot a_{w}\right)\left(1-b\cdot a_{w}+b\cdot c\cdot a_{w}\right)}$	Li et al. [9]
GDW	$M=\frac{a\cdot b\cdot a_{w}}{1+b\cdot a_{w}}\cdot \frac{1-c\cdot \left(1-d\right)\cdot a_{w}}{1-c\cdot a_{w}}$	Furmaniak, et al. [17]
Boquet	$M=\frac{a_{w}}{a+b\cdot a_{w}+c\cdot a_{w}^{2}}$	Liu, et al. [15]
MCPE	$a_{w}=\exp\left[\frac{-a\cdot \exp\left(-c\cdot M\right)}{b+t}\right]$ $M=\frac{1}{-c}\ln\left[\frac{\left(t+b\right)\ln\left(a_{w}\right)}{-a}\right]$	Li, et al. [9]
MGAB	$M=\frac{a\cdot b\cdot \left(c/t\right)\cdot a_{w}}{\left(1-b\cdot a_{w}\right)\left(1-b\cdot a_{w}+b\cdot \left(c/t\right)\cdot a_{w}\right)}$ $a_{w}=\frac{2+\frac{c}{t}\left(\frac{a}{M}-1\right)-{\left[{\left(2+\frac{c}{t}\left(\frac{a}{M}-1\right)\right)}^{2}-4\left(1-\frac{c}{t}\right)\right]}^{0.5}}{2b\left(1-\frac{c}{t}\right)}$	Cao, et al. [18]
Peleg	$M=a\cdot {a_{w}}^{c}+b\cdot {a_{w}}^{d}$	Liu, et al. [15]

Note: *M* is equilibrium moisture content (% w.b.), *a*_w_ is water activity (decimal), *t* is temperature (°C). a, b, c, and d are the equation parameters. GAB, Guggenheim–Anderson–de Boer; GDW, Generalized D’Arcy and Watt; MCPE, the modified Chung–Pfost; MGAB, the modified GAB.

**Table 3:** The fitting results for the moisture adsorption isotherms of FOS and inulin samples.

Equation	Samples	Equation	Parameters		Statistical	Parameters		
		a	b	c	*RSS*	*SE*	*R* ^2^	*MRE* (%)
Boquet	FOS1	5.824 × 10^−2^	2.022 × 10^−2^	−6.754 × 10^−2^	75.65	2.3873	0.9849	13.7316
	FOS2	9.647 × 10^−2^	−7.920 × 10^−2^	−1.135 × 10^−2^	111.3702	3.3749	0.9891	12.4943
	INU1	4.020 × 10^−2^	6.256 × 10^−2^	−8.209 × 10^−2^	68.4299	2.0735	0.9739	12.4264
	INU2	4.131 × 10^−2^	5.890 × 10^−2^	−7.953 × 10^−2^	62.5661	1.8959	0.9764	12.2518
	INU3	7.397 × 10^−2^	−1.966 × 10^−2^	−4.674 × 10^−2^	78.6517	2.3834	0.9894	10.8291
	FOS-aver	4.603 × 10^−2^	7.125 × 10^−2^	−9.184 × 10^−2^	58.9874	1.7875	0.9767	13.3542
	INU-aver	4.072 × 10^−2^	6.405 × 10^−2^	−8.433 × 10^−2^	61.4895	1.8633	0.9763	12.4709
Ferro–Fontan	FOS1	1.176	4.984	0.785	71.3189	2.1622	0.9864	10.3899
	FOS2	1.152	3.608	0.666	101.2958	3.0696	0.9901	9.7479
	INU1	1.312	6.237	0.826	69.5126	2.1064	0.9735	10.4663
	INU2	1.326	6.089	0.811	63.6629	1.9292	0.9759	7.9618
	INU3	1.151	4.254	0.737	70.8449	2.1468	0.9905	7.7725
	FOS-aver	1.262	6.225	0.861	56.4328	1.7101	0.9777	11.1907
	INU-aver	1.298	6.192	0.833	61.3395	1.8587	0.9764	10.4688
GAB	FOS1	5.045	0.978	−4.04 × 10^8^	201.3446	5.1014	0.9615	30.695
	FOS2	5.153	1.011	−2.90 × 10^8^	368.3782	11.1629	0.9637	50.5032
	INU1	7.644	0.849	3.83 × 10^0^	68.4299	2.0736	0.9739	12.4264
	INU2	7.759	0.847	3.68 × 10^0^	62.5661	1.8959	0.9764	12.2518
	INU3	5.071	0.998	−3.37 × 10^8^	256.0895	7.7604	0.9656	37.5305
	FOS-aver	7.071	0.864	4.03 × 10^0^	58.9874	1.7875	0.9767	13.3542
	INU-aver	7.485	0.853	3.84 × 10^0^	61.4895	1.8633	0.9764	12.4709
MGAB	FOS1	9.397	0.892	42.419	54.9115	1.6639	0.9895	15.4482
	FOS2	11.008	0.933	23.839	65.9337	1.9979	0.9935	15.0965
	INU1	9.088	0.814	69.466	50.1801	1.5206	0.9809	12.7239
	INU2	8.795	0.822	74.075	47.1707	1.4294	0.9822	12.4161
	INU3	9.999	0.918	31.607	45.0625	1.3655	0.9939	13.9382
	FOS-aver	7.507	0.852	91.862	51.1411	1.5497	0.9798	13.7313
	INU-aver	8.392	0.831	78.094	48.0215	1.4552	0.9815	12.7838

Note: Boquet, Ferro–Fontan, GAB and MGAB are in a form of $M=f\left(a_{w},t\right)$; GAB, Guggenheim–Anderson–de Boer; MGAB, the modified GAB; a, b, and c are the equation parameters; FOS-aver is the mean adsorptive EMC/*a*_w_ data of two FOS samples; INU-aver is the mean adsorptive EMC/*a*_w_ data of three inulin samples; *RSS* residue sum of squares; *SE*, standard error; *R*^2^, determination coefficient; *MRE*, mean relative percentage error.

**Table 4:** The fitting results for the moisture desorption isotherms of FOS and inulin samples.

Equation	Samples	Equation	Parameters		Statistical	Parameters		
		a	b	c	*RSS*	*SE*	*R* ^2^	*MRE* (%)
Boquet	FOS1	6.041 × 10^−3^	1.380 × 10^−1^	−1.345 × 10^−1^	85.3299	2.5858	0.9805	9.7775
	FOS2	−4.772 × 10^−3^	1.368 × 10^−1^	−1.264 × 10^−1^	194.9933	5.9089	0.9717	9.382
	INU1	1.129 × 10^−3^	1.558 × 10^−1^	−1.377 × 10^−1^	41.2975	1.2513	0.9784	5.4797
	INU2	1.075 × 10^−3^	1.569 × 10^−1^	−1.389 × 10^−1^	39.8948	1.2089	0.9793	5.6076
	INU3	−2.422 × 10^−3^	1.451 × 10^−1^	−1.355 × 10^−1^	120.7191	3.6582	0.9782	9.3627
	FOS-aver	1.869 × 10^−3^	1.584 × 10^−1^	−1.411 × 10^−1^	29.2268	0.8857	0.9846	4.2232
	INU-aver	1.342 × 10^−3^	1.571 × 10^−1^	−1.392 × 10^−1^	36.2956	1.0998	0.9811	5.0535
Ferro–Fontan	FOS1	1.026	22.381	1.378	86.2854	2.6147	0.9803	9.6767
	FOS2	0.899	131.519	1.979	253.9399	7.6951	0.9235	12.9858
	INU1	0.998	82.488	1.957	39.8588	1.2078	0.9792	5.4127
	INU2	0.996	82.828	1.962	38.1094	1.1548	0.9803	5.5501
	INU3	0.966	92.038	1.843	152.1041	4.6092	0.9725	10.0406
	FOS-aver	1.006	62.711	1.863	28.7871	0.8723	0.9848	4.2237
	INU-aver	1.000	75.336	1.927	35.1198	1.0642	0.9816	4.9664
GAB	FOS1	6.679	0.934	26.536	85.3299	2.5858	0.9805	9.7775
	FOS2	8.493	0.941	5.42 × 10^8^	268.8505	8.1469	0.9609	11.9103
	INU1	6.336	0.878	159.284	41.2935	1.2513	0.9784	5.4797
	INU2	6.298	0.879	167.825	39.8948	1.2089	0.9793	5.6076
	INU3	7.365	0.943	3.15 × 10^8^	128.2907	3.8876	0.9768	9.2657
	FOS-aver	6.182	0.881	98.267	29.2269	0.8857	0.9846	4.2232
	INU-aver	6.271	0.879	135.115	36.2956	1.0998	0.9811	5.0538
MGAB	FOS1	6.913	0.928	465.662	74.6088	2.2609	0.9829	8.8713
	FOS2	8.493	0.941	4.22 × 10^9^	268.8509	8.1469	0.9609	11.9103
	INU1	6.458	0.873	2035.817	39.4308	1.1949	0.9794	4.8227
	INU2	6.421	0.875	2073.621	38.0729	1.1537	0.9803	4.9639
	INU3	7.365	0.943	1.64 × 10^9^	128.2907	3.8876	0.9768	9.2657
	FOS-aver	6.273	0.877	1720.989	27.4963	0.8332	0.9855	3.6035
	INU-aver	6.383	0.875	1935.73	34.4638	1.0444	0.9819	4.3433

Note: Boquet, Ferro–Fontan, GAB and MGAB are in a form of $M=f\left(a_{w},t\right)$; GAB, Guggenheim–Anderson–de Boer; MGAB, the modified GAB; a, b, and c are the equation parameters; FOS-aver is the mean desorptive EMC/*a*_w_ data of two FOS samples; INU-aver is the mean desorptive EMC/*a*_w_ data of three inulin samples; *RSS*, residue sum of squares; *SE*, standard error; *R*^2^, determination coefficient; *MRE*, mean relative percentage error.

**Table 5:** Fitting the moisture sorption isotherms of FOS and inulin samples using GDW and Peleg.

Equation	Sorption	Samples	Equation	Parameters			Statistical Parameters			
			a	b	c	d	*RSS*	*SE*	*R* ^2^	*MRE* (%)
GDW	Ads	FOS1	1.624	−1.011 × 10^10^	0.893	5.985	70.0397	2.1887	0.9866	12.9
		FOS2	0.638	8.091 × 10^13^	0.947	15.145	107.4391	3.3575	0.9894	10.302
		INU1	1.939	3.201 × 10^15^	0.777	6.173	69.0347	2.1573	0.9737	9.577
		INU2	1.884	−3.402 × 10^15^	0.775	6.445	63.0002	1.9688	0.9762	9.4799
		INU3	1.176	−5.219 × 10^14^	0.927	8.073	71.8381	2.2449	0.9904	7.7725
		FOS-aver	2.026	−1.432 × 10^15^	0.802	5.227	55.5323	1.7354	0.9781	10.3702
		INU-aver	1.954	5.358 × 10^15^	0.785	5.904	60.7027	1.8969	0.9766	9.5277
GDW	Des	FOS1	5.079	3.479 × 10^13^	0.911	1.573	82.4221	2.5757	0.9812	9.7117
		FOS2	10.716	1.895 × 10^14^	0.986	0.538	199.9668	6.2489	0.9709	9.3569
		INU1	6.284	−3.539 × 10^14^	0.885	0.973	41.8334	1.3073	0.9782	5.8434
		INU2	6.276	−9.768 × 10^13^	0.888	0.962	40.3433	1.2607	0.9791	5.9754
		INU3	8	−6.846 × 10^14^	0.958	0.815	123.1143	3.8473	0.9777	9.3528
		FOS-aver	5.901	−7.167 × 10^13^	0.881	1.061	30.3986	0.9499	0.9839	4.6303
		INU-aver	6.154	−2.450 × 10^13^	0.885	0.997	37.0555	1.1579	0.9806	5.4111
Peleg	Ads	FOS1	14.673	49.755	0.837	5.572	80.9986	2.5312	0.9845	12.5587
		FOS2	16.396	84.128	1.011	6.731	92.4257	2.8883	0.9909	11.4296
		INU1	24.366	40.734	1.219	14.457	64.0573	2.0018	0.9756	14.2233
		INU2	24.698	41.827	1.237	14.854	58.0427	1.8138	0.9781	14.1289
		INU3	16.466	66.614	0.971	6.441	73.3775	2.2931	0.9902	10.4121
		FOS-aver	22.691	33.375	1.194	11.601	61.0083	1.9065	0.9759	15.7294
		INU-aver	24.021	38.471	1.222	13.708	59.2958	1.8529	0.9772	14.606
Peleg	Des	FOS1	14.172	50.057	0.424	5.635	87.4058	2.7304	0.9801	9.7695
		FOS2	15.045	80.266	0.08883	6.352	166.8639	5.2145	0.9758	9.2847
		INU1	13.804	31.688	0.358	5.943	40.3411	1.2607	0.9789	5.4666
		INU2	13.848	32.135	0.362	6.035	38.9284	1.2165	0.9798	5.5801
		INU3	14.222	65.268	0.209	5.998	118.1249	3.6914	0.9786	9.4316
		FOS-aver	13.401	31.104	0.369	5.796	29.1831	0.9124	0.9846	4.3630
		INU-aver	13.685	31.636	0.363	5.924	35.693	1.1154	0.9813	5.0454

Note: GDW and Peleg are in a form of $M=f\left(a_{w},t\right)$; GDW, Generalized D’Arcy and Watt; a, b, c, and d are the equation parameters; FOS-aver is the mean adsorptive or desorptive EMC/*a*_w_ data of two FOS samples; INU-aver is the mean adsorptive or desorptive EMC/*a*_w_ data of three inulin samples; Ads, adsorption; Des, desorption; *RSS*, residue sum of squares; *SE*, standard error; *R*^2^, determination coefficient; *MRE*, mean relative percentage error.

**Table 6:** The fitting results for the moisture sorption isotherms of FOS and inulin samples using a Polynomial.

Sorption	Samples	Equation	Parameters						Statistical	Parameters
		a	b	c	d	e	f	g	*R* ^2^	*MRE* (%)
Ads	FOS1	22.263	−2.0714	6.41 × 10^−2^	−6.80 × 10^−4^	36.532	−98.016	116.264	0.9851	12.9000
	FOS2	−63.883	6.691	−2.48 × 10^−1^	2.93 × 10^−3^	99.468	−276.555	268.853	0.9882	24.3912
	INU1	−20.728	2.767	−1.09 × 10^−1^	1.33 × 10^−3^	20.955	−31.751	48.274	0.9758	11.9344
	INU2	−19.854	2.619	−1.02 × 10^−1^	1.23 × 10^−3^	20.257	−29.928	47.211	0.9773	11.5841
	INU3	−20.811	2.309	−9.18 × 10^−2^	7.12 × 10^−3^	67.999	−187.285	194.058	0.9881	16.4874
	FOS-aver	15.452	−1.33	4.41 × 10^−2^	−5.09 × 10^−4^	14.83	−23.366	44.852	0.9768	11.2958
	INU-aver	−8.379	1.352	−5.54 × 10^−2^	6.87 × 10^−4^	18.681	−28.347	46.779	0.9771	11.4668
Des	FOS1	−89.641	10.631	−3.96 × 10^−1^	4.72 × 10^−3^	56.274	−145.635	146.771	0.9911	6.0063
	FOS2	−76.452	10.133	−3.91 × 10^−1^	4.75 × 10^−3^	84.953	−263.627	255.919	0.9897	5.7773
	INU1	−12.433	2.081	−8.55 × 10^−2^	1.07 × 10^−3^	45.291	−106.639	100.212	0.9888	3.8617
	INU2	−14.5	2.309	−9.40 × 10^−2^	1.17 × 10^−3^	45.966	−108.619	101.813	0.9894	3.8712
	INU3	−83.046	10.382	−3.93 × 10^−1^	4.73 × 10^−3^	70.614	−204.631	201.345	0.9911	5.6422
	FOS-aver	18.026	−1.439	4.47 × 10^−2^	−4.90 × 10^−4^	43.723	−102.515	97.256	0.9905	3.6893
	INU-aver	−2.969	0.9837	−4.49 × 10^−2^	5.82 × 10^−4^	44.993	−105.925	99.761	0.9896	3.8198

Note: FOS-aver is the mean adsorptive or desorptive EMC/*a*_w_ data of two FOS samples; INU-aver is the mean adsorptive or desorptive EMC/*a*_w_ data of three inulin samples; Ads, adsorption; Des, desorption; a, b, c, d, e, f, and g are the Polynomial parameters; *R*^2^, determination coefficient; *MRE*, mean relative percentage error.

**Table 7:** Fitting the moisture sorption isotherms of FOS and inulin samples using MCPE.

Equation	Sorption	Samples	Equation	Parameters		Statistical	Parameters		
			a	b	c	*RSS*	*SE*	*R* ^2^	*MRE* (%)
$M=f\left(a_{w},t\right)$	Ads	FOS1	192.973	74.547	8.07 × 10^−2^	441.1844	13.3692	0.9155	42.2929
		FOS2	188.219	90.979	5.93 × 10^−2^	1271.344	38.5256	0.8745	79.0006
		INU1	242.255	75.762	1.14 × 10^−1^	127.2049	3.8547	0.9498	23.3271
		INU2	171.271	44.641	1.16 × 10^−1^	109.6001	3.3212	0.9581	20.7637
		INU3	188.458	52.262	1.11 × 10^−1^	107.1159	3.2459	0.9595	20.9011
		FOS-aver	189.842	83.496	6.84 × 10^−2^	782.67	23.7173	0.8949	56.9353
		INU-aver	195.948	55.488	1.12 × 10^−1^	112.9711	3.4234	0.9565	21.3975
	des	FOS1	133.035	25.277	8.88 × 10^−2^	383.6574	11.6259	0.9123	24.3588
		FOS2	127.447	12.401	7.52 × 10^−2^	1278.414	38.7398	0.8145	26.5989
		INU1	234.023	36.341	1.33 × 10^−1^	119.804	3.6304	0.9368	13.5253
		INU2	197.597	24.249	1.33 × 10^−1^	126.4941	3.8332	0.9339	13.0525
		INU3	195.988	24.285	1.32 × 10^−1^	128.4974	3.8939	0.9334	13.2478
		FOS-aver	128.783	17.439	8.14 × 10^−2^	758.0744	22.9719	0.8628	25.2102
		INU-aver	207.376	27.713	1.33 × 10^−1^	124.6282	3.7766	0.9349	13.2529
$a_{w}=f\left(M,t\right)$	Ads	FOS1	692.292	303.719	9.99 × 10^−2^	0.1419	4.30 × 10^−3^	0.9409	22.4137
		FOS2	376.423	168.041	9.33 × 10^−2^	0.1659	5.03 × 10^−3^	0.9309	19.893
		INU1	708.351	268.984	1.21 × 10^−1^	0.1025	3.11 × 10^−3^	0.9573	18.2084
		INU2	342.882	113.251	1.19 × 10^−1^	0.07214	2.19 × 10^−3^	0.9699	15.8039
		INU3	374.672	127.417	1.18 × 10^−1^	0.07104	2.15 × 10^−3^	0.9704	15.7335
		FOS-aver	501.712	222.593	9.65 × 10^−2^	0.1457	4.41 × 10^−3^	0.9393	20.7755
		INU-aver	431.559	150.914	1.19 × 10^−1^	0.07894	2.39 × 10^−3^	0.9671	16.3496
	Des	FOS1	181.483	28.762	1.18 × 10^−1^	0.1279	3.88 × 10^−3^	0.9467	19.294
		FOS2	225.796	−0.09954	1.45 × 10^−1^	0.3211	3.73 × 10^−3^	0.8662	30.5305
		INU1	431.376	57.777	1.70 × 10^−1^	0.06699	2.02 × 10^−3^	0.9721	12.6296
		INU2	332.974	33.861	1.71 × 10^−1^	0.06377	1.93 × 10^−3^	0.9734	11.9331
		INU3	324.541	32.561	1.71 × 10^−1^	0.06436	1.95 × 10^−3^	0.9732	11.9689
		FOS-aver	188.187	10.805	1.29 × 10^−1^	0.2074	6.28 × 10^−3^	0.9136	24.2068
		INU-aver	355.421	39.596	1.71 × 10^−1^	0.06454	1.96 × 10^−3^	0.9731	12.1971

Note: FOS-aver is the mean adsorptive or desorptive EMC/*a*_w_ data of two FOS samples; INU-aver is the mean adsorptive or desorptive EMC/*a*_w_ data of three inulin samples; Ads, adsorption; Des, desorption; a, b, and c are MCPE parameters; *RSS*, residue sum of squares; *SE*, standard error; *R*^2^, determination coefficient; *MRE*, mean relative percentage error.

**Table 8:** Determination of the best-fitting equations for FOS and inulin.

Sorption	Equation	Statistical	Parameters			Order
		RSS	SE	R^2^	MRE (%)	
Ads	Boquet	73.8778 ± 18.0944	2.2523 ± 10.5511	0.9809 ± 0.0066	12.5083 ± 0.9225	3
	Ferro–Fontan	70.6296 ± 14.5978	2.1404 ± 0.4423	0.9815 ± 0.0072	9.7139 ± 1.3301	1
	GAB	153.8979 ± 123.7536	4.5207 ± 3.7049	0.9706 ± 0.0067	24.1760 ± 15.5366	7
	GDW	71.0838 ± 17.0448	2.2214 ± 0.5326	0.9816 ± 0.0069	9.9899 ± 1.5423	2
	MCPE	421.7273 ± 452.9505	12.7796 ± 13.7258	0.9298 ± 0.0348	37.8026 ± 22.8697	8
	MGAB	51.7744 ± 6.9952	1.5689 ± 0.2119	0.9859 ± 0.0062	13.7340 ± 1.1900	5
	Polynomial	75.4104 ± 22.3768	2.6003 ± 0.7716	0.9812 ± 0.0057	14.2942 ± 4.8063	6
	Peleg	69.8866 ± 12.9674	2.1839 ± 0.4053	0.9818 ± 0.0067	13.2983 ± 1.8945	4
Des	Boquet	78.2510 ± 61.2138	2.3712 ± 1.8549	0.9791 ± 0.0039	6.9837 ± 2.4055	4
	Ferro–Fontan	90.6006 ± 84.3703	2.7454 ± 2.5567	0.9717 ± 0.0216	7.5509 ± 3.3312	6
	GAB	89.8831 ± 86.5407	2.7237 ± 2.6224	0.9774 ± 0.0077	7.3311 ± 2.9422	7
	GDW	79.3049 ± 62.6993	2.4782 ± 1.9594	0.9788 ± 0.0041	7.1831 ± 2.1885	5
	MCPE	417.0814 ± 447.3834	12.6388 ± 13.5571	0.9041 ± 0.0475	18.4638 ± 6.5125	8
	MGAB	87.3163 ± 87.4499	2.6459 ± 2.6499	0.9782 ± 0.0081	6.8258 ± 3.1629	2
	Polynomial	34.1896 ± 20.1132	1.1789 ± 0.6935	0.9900 ± 0.0009	4.6668 ± 1.0749	1
	Peleg	73.7915 ± 52.5818	2.3059 ± 1.6431	0.9799 ± 0.0027	6.9916 ± 2.3785	3

Note: Ads, adsorption; Des, desorption; GAB, Guggenheim–Anderson–de Boer; GDW, Generalized D’Arcy and Watt; MCPE, the modified Chung–Pfost; MGAB, the modified GAB; *RSS*, residue sum of squares; *SE*, standard error; *R*^2^, determination coefficient; *MRE*, mean relative percentage error.

**Table 9:** The comparison of the absorbance values of main peaks in FTIR of FOS and inulin.

Sample		Absorbance	Values	
	514 cm^−1^	556 cm^−1^	749 cm^−1^	814 cm^−1^
FOS2	0.2675 ± 0.0075		0.1926 ± 0.0038	-
INU3	-	0.3082 ± 0.0035	-	0.2338 ± 0.0034
Sample	918 cm^−1^	925 cm^−1^	1020 cm^−1^	1084 cm^−1^
FOS2	0.2820 ± 0.0050	-	0.4101 ± 0.0072	0.3556 ± 0.0058
INU3	-	0.3525 ± 0.0045	0.6609 ± 0.0057	0.5216 ± 0.0061
Sample	1232 cm^−1^	1329 cm^−1^	1405 cm^−1^	1628 cm^−1^
FOS2	0.2216 ± 0.0025	-	0.2317 ± 0.0025	0.1342 ± 0.0022
INU3	-	0.2968 ± 0.0060	-	0.2046 ± 0.0062
Sample	2072 cm^−1^	2892 cm^−1^	2915 cm^−1^	3337 cm^−1^
FOS2	0.0460 ± 0.0069	0.1987 ± 0.0044	-	0.3559 ± 0.0033
INU3	0.1057 ± 0.0100	-	0.2913 ± 0.0104	0.6138 ± 0.0117

Note: - shows no clear peak.

**Table 10:** Effect of adding FOS or inulin on the pasting parameters of rice starch.

Addition	Peak Viscosity (cp)	Trough Viscosity(cp)	Breakdown Viscosity (cp)	Final Viscosity (cp)	Setback Viscosity (cp)	Peak Time (min)	Pasting Temp. (°C)
0%	2724 ± 24 ^a^	2117 ± 10 ^a^	609 ± 21 ^a^	3001 ± 22 ^a^	885 ± 19 ^a^	6.44 ± 0.07 ^e^	75.43 ± 0.01 ^c^
3% FOS2	2539 ± 34 ^b^	1967 ± 68 ^c^	573 ± 50 ^a^	2839 ± 32 ^b^	872 ± 43 ^ab^	6.47 ± 0.14 ^de^	74.77 ± 0.49 ^d^
5% FOS2	2378 ± 35 ^c^	1954 ± 103 ^c^	424 ± 81 ^bc^	2702 ± 20 ^c^	748 ± 93 ^bcde^	6.67 ± 0.14 ^bcd^	75.57 ± 0.45 ^bcd^
7% FOS2	2228 ± 10 ^d^	1801 ± 48 ^d^	427 ± 38 ^bc^	2568 ± 5 ^d^	767 ± 44 ^cd^	6.58 ± 0.10 ^de^	75.30 ± 0.48 ^bcd^
10% FOS2	2019 ± 6 ^f^	1689 ± 3 ^e^	330 ± 3 ^d^	2371 ± 3 ^f^	682 ± 2 ^e^	6.67 ± 0.07 ^cd^	76.13 ± 0.45 ^b^
3% INU3	2527 ± 14 ^b^	2107 ± 7 ^b^	422 ± 21 ^b^	2855 ± 3 ^b^	749 ± 10 ^c^	6.75 ± 0.04 ^bc^	75.60 ± 0.48 ^bcd^
5% INU3	2357 ± 14 ^c^	1996 ± 22 ^c^	361 ± 36 ^cd^	2699 ± 5 ^c^	703 ± 23 ^de^	6.75 ± 0.04 ^bc^	75.88 ± 0.03 ^b^
7% INU3	2157 ± 1 ^e^	1778 ± 30 ^d^	378 ± 29 ^bc^	2502 ± 6 ^e^	723 ± 34 ^cd^	6.84 ± 0.08 ^b^	76.07 ± 0.46 ^b^
10% INU3	1918 ± 8 ^g^	1502 ± 9 ^f^	416 ± 8 ^b^	2243 ± 9 ^g^	740 ± 8 ^c^	6.98 ± 0.04 ^a^	76.63 ± 0.03 ^a^

Note: All data are expressed as mean ± SD; number of repetitions—*n* = 3. Different superscript letters indicate significant differences (*p* < 0.05) within the column.

**Table 11:** Effect of adding FOS or inulin on the thermal properties of rice starch samples.

Addition	Δ*H* (J/g)	*T*_o_ (°C)	*T*_p_ (°C)	*T*_c_ (°C)	Peak Width (°C)	Peak Height (0.01 mW/mg)	Aging (%)
0%	9.54 ± 0.10 ^e^	62.95 ± 1.16 ^abcde^	69.85 ± 0.94 ^a^	77.87 ± 1.08 ^abc^	7.70 ± 0.23 ^abc^	16.16 ± 0.45 ^bcd^	38.20 ± 2.60 ^b^
3% FOS2	9.71 ± 0.06 ^d^	61.97 ± 0.31 ^e^	69.17 ± 0.15 ^c^	77.37 ± 0.67 ^c^	7.43 ± 0.15 ^c^	17.14 ± 0.34 ^a^	29.39 ± 0.70 ^c^
5% FOS2	9.85 ± 0.01 ^c^	62.15 ± 0.15 ^de^	69.45 ± 0.25 ^bc^	77.90 ± 0.70 ^bc^	7.60 ± 0.30 ^bc^	17.01 ± 0.63 ^abc^	26.12 ± 0.76 ^d^
7% FOS2	9.95 ± 0.02 ^b^	62.53 ± 0.25 ^d^	69.70 ± 0.17 ^b^	78.07 ± 0.47 ^bc^	7.53 ± 0.35 ^bc^	17.16 ± 0.81 ^abc^	20.44 ± 0.97 ^e^
10% FOS2	10.11 ± 0.03 ^a^	63.03 ± 0.23 ^c^	70.50 ± 0.26 ^a^	79.23 ± 0.32 ^a^	7.70 ± 0.17 ^bc^	16.82 ± 0.32 ^ab^	15.55 ± 0.54 ^f^
3% INU3	9.21 ± 0.09 ^f^	64.10 ± 0.14 ^a^	70.70 ± 0.42 ^a^	78.25 ± 0.49 ^bc^	7.75 ± 0.07 ^b^	15.70 ± 0.17 ^d^	43.62 ± 0.15 ^a^
5% INU3	9.70 ± 0.17 ^cde^	63.80 ± 0.44 ^ab^	70.77 ± 0.25 ^a^	77.70 ± 0.36 ^c^	7.83 ± 0.12 ^ab^	16.10 ± 0.32 ^cd^	35.17 ± 1.95 ^b^
7% INU3	9.76 ± 0.34 ^abcde^	63.63 ± 0.06 ^b^	70.80 ± 0.10 ^a^	78.37 ± 0.51 ^bc^	7.97 ± 0.06 ^a^	16.20 ± 0.54 ^bcd^	33.68 ± 2.80 ^b^
10% INU3	8.57 ± 0.17 ^g^	64.17 ± 0.21 ^a^	71.03 ± 0.47 ^a^	78.70 ± 0.44 ^ab^	7.63 ± 0.15 ^c^	15.00 ± 0.26 ^e^	33.07 ± 7.13 ^bcd^

Note: Δ*H*, Gelatinization enthalpy; *T*_o_, *T*_p_, and *T*_c_ are the onset temperature, peak temperature, and conclusion temperature of gelatinization, respectively. All data are expressed as mean ± SD; number of repetitions—*n* = 3. Different superscript letters indicate significant differences (*p* < 0.05) within the column.

**Table 12:** Influence of FOS or inulin on the thermal properties of rice starch using General Linear Model (GLM) analysis.

Factors	Levels	Δ*H* (J/g)	*T*_o_ (°C)	*T*_p_ (°C)	*T*_c_ (°C)	Peak Width (°C)	Peak Height (0.01 mW/mg)	Aging (%)
Species	FOS2	9.81 ± 0.091 ^a^	62.32 ± 0.09 ^d^	69.58 ± 0.09 ^d^	77.96 ± 0.17 ^bc^	7.59 ± 0.06 ^c^	16.82 ± 0.16 ^a^	25.79 ± 1.04 ^c^
	INU3	9.37 ± 0.09 ^c^	63.940 ± 0.09 ^a^	70.78 ± 0.09 ^a^	78.30 ± 0.17 ^b^	7.78 ± 0.06 ^a^	15.87 ± 0.16 ^d^	36.90 ± 1.04 ^a^
Addition	0	9.54 ± 0.15 ^bc^	62.95 ± 0.14 ^c^	69.85 ± 0.14 ^c^	77.87 ± 0.27 ^b^	7.70 ± 0.09 ^abc^	16.16 ± 0.25 ^cd^	38.21 ± 1.64 ^a^
(%)	3	9.46 ± 0.15 ^c^	63.03 ± 0.14 ^c^	69.94 ± 0.14 ^c^	77.81 ± 0.27 ^c^	7.59 ± 0.09 ^bc^	16.41 ± 0.25 ^bc^	36.51 ± 1.64 ^a^
	5	9.77 ± 0.15 ^ab^	62.98 ± 0.14 ^c^	70.11 ± 0.14 ^bc^	77.80 ± 0.27 ^c^	7.72 ± 0.09 ^abc^	16.54 ± 0.25 ^abc^	30.64 ± 1.64 ^b^
	7	9.85 ± 0.15 ^a^	63.09 ± 0.14 ^c^	70.25 ± 0.14 ^b^	78.22 ± 0.27 ^bc^	7.75 ± 0.09 ^ab^	16.70 ± 0.25 ^ab^	27.06 ± 1.64 ^c^
	10	9.34 ± 0.15 ^c^	63.60 ± 0.14 ^b^	70.77 ± 0.14 ^a^	78.97 ± 0.27 ^a^	7.67 ± 0.09 ^abc^	15.90 ± 0.25 ^d^	24.31 ± 1.64 ^c^

Note: Δ*H*, Gelatinization enthalpy; *T*_o_, *T*_p_, and *T*_c_ are the onset temperature, peak temperature, and conclusion temperature of gelatinization, respectively. All data are expressed as mean ± SD; number of repetitions—*n* = 3. Different superscript letters indicate significant differences (*p* < 0.05) within the column.

## Data Availability

The original contributions presented in this study are included in this article;the raw EMC–a_w_ matrices, fits, code, and other inquiries can be directed to the corresponding author.
